# Curdlan enhances the structure of myosin gel model

**DOI:** 10.1002/fsn3.1055

**Published:** 2019-05-01

**Authors:** Qianru Li, Peisen Wang, Song Miao, Longtao Zhang, Baodong Zheng

**Affiliations:** ^1^ College of Food Science Fujian Agriculture and Forestry University Fuzhou China; ^2^ China‐Ireland International Cooperation Centre for Food Material Science and Structural Design Fujian Agriculture and Forestry University Fuzhou China; ^3^ Fujian Provincial Key Laboratory of Quality Science and Processing Technology in Special Starch Fujian Agriculture and Forestry University Fuzhou China; ^4^ Teagasc Food Research Centre Moorepark, Fermoy, Co. Cork Ireland

**Keywords:** curdlan, microstructure, myosin gel, physiochemical property

## Abstract

The aim of this work was to investigate the gelation mechanism of curdlan on surimi using a myosin gel model. Experimental results showed that with increased levels of curdlan, the water‐holding capacity, gel strength, and storage modulus of a myosin gel first increased and then decreased. The optimum level of curdlan was found to be 1%. Moreover, myosin–curdlan mixed gel showed decreased water liquidity based on the results of low‐field nuclear magnetic resonance. The enhanced physicochemical properties of myosin–curdlan mixed gel were attributed to the strengthened hydrogen bonding and to the uniform and compact network structure shown by Fourier‐transform infrared spectroscopy and scanning electron microscopy. The results of this study suggest that curdlan has the potential to be used in surimi‐based seafood products to enhance the gel structure.

## 1 INTRODUCTION

Surimi gel, prepared from deboned and washed fish paste, has been commonly used to produce restructured gel‐based seafood products due to its unique textural properties and high nutritional value. In the food industry, water‐holding capacity (WHC), gel strength, and some other physicochemical properties of surimi products are always difficult to maintain because of the unavoidable environmental factors during refrigeration, transportation, and sale. That affects the quality of surimi products. Hydrophilic colloids are added to improve the stability of surimi products during circulation (Ayadi, Kechaou, Makni, & Attia, 2009; Chin, Go, & Xiong, 2009).

Curdlan is a linear, nonbranched hydrophilic colloid consisting of β‐1,3‐glycosidic bonds. Its suspensions may form thermally irreversible gels when heated to 80°C. In a thermally irreversible gel, the curdlan micelles are cross‐linked with molecules of a single helix using hydrogen bonds and cross‐linked with triple‐stranded helices using hydrophobic interactions (Funami, Yada, & Nakao, 1998; Kanzawa, Harada, Koreeda, Harada, & Okuyama, 1989). Due to curdlan's unique thermal gel properties, foundational research into its use has been widespread and applied in the food industry. Funami et al. (1998) studied the effects of curdlan on the thermal characteristics, gel properties, and rheological properties of pork meat. The results showed that pork–curdlan mixed gel formed a strong gel structure that entrapped significant amounts of water within the gel structure and improved its WHC. Hu et al. (2015) and Wu et al. (2015) studied the effect of curdlan on surimi and found that its addition improved the gel properties of the muscle protein of ribbonfish. That was also indicated in the study of Wei et al. (2018). The interaction mechanism between curdlan and surimi protein was also discussed. According to the results of dynamic rheology and differential scanning calorimetry, during the heating process, the addition of curdlan did not influence the denaturation of myosin but did affect the interaction with actin and increased its thermal transition temperature (Wei et al., 2018). Surimi is a complex protein gel system. Myosin is the main gelation protein in fish muscles that is responsible for the formation of surimi (Chan, Gill, & Paulson, 2010; Tao, Kobayashi, Fukushima, & Watabe, 2010). Thus far, the effect of curdlan on the single myosin gel model has not been reported.

To investigate the mechanism of curdlan application on surimi, a myosin gel model is used. The effect of curdlan at different concentrations on the physicochemical properties of myosin gels, such as gel strength, WHC, and the rheology properties, is investigated. The status of water in the myosin gel is further revealed by low‐field nuclear magnetic resonance (LF‐NMR). The interaction between myosin–curdlan mixed gels systems is here characterized using Fourier‐transform infrared spectroscopy (FT‐IR), and the microstructure is reflected by scanning electron microscopy (SEM).

## 2 MATERIALS AND METHODS

### 2.1 Materials and chemicals

Fresh tilapia (500 g ± 100 g) was purchased from the Yonghui supermarket (a local supermarket in Fuzhou, China). Curdlan was purchased from VWR Life Science Amresco Products, Avantor Performance Materials, Inc. Tris, HCl, KCl, NaN_3_, β‐mercaptoethanol, Mg (CH_3_COO)_2_, ethylene glycol‐bis‐(2‐chloroethyl) tetraacetic acid (EGTA), ATPNa_2_, KHCO_3_, MgCl_2_, formaldehyde, glutaraldehyde, phosphoric acid salt buffer, ethanol, and acetone were obtained from Solarbio Science & Technology Co., Ltd. The above reagents were all of analytical grade.

### 2.2 Preparation of myosin

Myosin was prepared according to the method of Park and Lanier (1989) with minor modification. After the fish was peeled and boned, it was homogenized and washed in ten volumes of solution A (20 mM Tris–HCl buffer containing 0.1 mol/L KCl and 0.02% NaN_3_, pH 7.5) at 4°C for 15 min and centrifuged at 9,632 *g* for 10 min. The precipitate was suspended for one hour in five volumes of solution B (20 mM of Tris–HCl buffer containing 0.45 M KCl, 5 mM β‐mercaptoethanol, 0.2 M Mg (CH_3_COO)_2,_ and 1 mM EGTA, pH 6.8). ATPNa_2_ was then added to a final concentration of 5 mM, and the sample was centrifuged for 10 min at 9,632 *g*. The supernatant was diluted with five volumes of 1 mM KHCO_3_, incubated 15 min at 4°C, and centrifuged at 13,870 *g* for 10 min. The precipitate was then resuspended in 2.5 volumes of solution C (20 mM of Tris–HCl buffer containing 0.5 M KCl and 5 mM β‐mercaptoethanol, pH 7.5), incubated at 4°C for 10 min, and diluted with 2.5 volumes of 1 mM KHCO_3_. Afterward, MgCl_2_ was added to a final concentration of 10 mM, and the sample was incubated at 4°C overnight. After centrifugation (10,000 *g* for 15 min), the precipitate was resuspended with one volume of solution C, the myosin solution. The protein concentration was measured using the Biuret method, and the protein solution was adjusted to 40 mg/ml with a 0.6 M KCL solution.

### 2.3 Myosin gel preparation

Curdlan was added to the myosin solution at the following ratios (w/w) with stirring: 0, 0.25%, 0.5%, 0.75%, 1%, 1.25%, and 1.5% to create seven distinct samples. A pure myosin solution without curdlan was prepared as the control group. The sample solutions were homogenized at 10,000 rpm for 1 min and stored at 4°C overnight. Then, sample gels were formed by heating in a water bath at 80°C for 20 min (another 30 min were needed to warm the system from 20 to 80°C). After that, the samples were cooled immediately and stored at 4°C before measurement.

### 2.4 Water‐holding capacity of the gels

Water‐holding capacity was measured according to the method of Lopez‐Diaz, Rodriguez‐Romero, Hernandez‐Santoyo, Sotelo‐Mundo, and de la Barca (2003). The gels were centrifuged at 1,500 *g* for 10 min at 25°C. The WHC was calculated as follows:

(1) $$\text{WHC}\;\left(\%\right)=W_{2}/W_{1}$$

where *W*_1_ is the gel weight before centrifugation and *W*_2_ is the gel weight after centrifugation. Measurements were performed for all six samples and the control sample in parallel.

### 2.5 Gel strength measurements

The gel strength was measured according to the method published by Zhou et al. (2014). The gels were prepared and measured in a cylindrical container (30 mm × 45 mm). They were stored at 4°C for 24 hr and then equilibrated at room temperature for 30 min prior to measurement. The gel strength was measured using a TA. XT plus texture analyzer (Stable Micro System) using the GMIA Gelatine program compression mode test. The specifications of the compression mode test are as follows: probe model of P/0.5, pretest speed of 2 mm/s, test speed of 1 mm/s, posttest speed of 2 mm/s, a distance of 40% initial height, trigger force of 5 g, data acquisition speed of 200 pps, and an automatic trigger type. The measurements were performed in six replicates. Gel strength (g) is defined as the maximum force required for gel cracking.

### 2.6 Rheology analysis

A physical MCR 301 rotational rheometer (Anton Paar, GmbH‐Graz) equipped with a PP50 (0.55 mm gap) parallel plate was used to record the rheological properties. The storage modulus (G′) was recorded at 0.1 Hz and a 2% strain function of time. The temperature test was programmed to heat the samples from 20 to 80°C, and the heating rate was 2°C per minute.

### 2.7 Low‐field nuclear magnetic resonance

A low‐field pulsed NMI 20‐analyst (MesoMR23‐060H‐I NMR and MRI, Shanghai Niumag Electronic Technology Co., Ltd.) with 18.183 MHz was used according to the method of Zhang et al. (2016) to determine water migration within the myosin gel.

The typical pulse parameters recorded were as follows: spectrometer frequency (SF), 18.183 MHz; magnet strength, 0.43 T; pulses of 90° (P90), 5 μs; pulses of 180° (P180), 10.4 μs; scan repetitions (NS), 4; echo count (NECH), 12,000; test wait time (TW), 8,000 ms; and echo time (TE), 0.8 ms.

### 2.8 Fourier‐transform infrared spectroscopy

The samples were freeze‐dried for potassium bromide tableting and placed in a Fourier sample cell for testing. The Fourier spectral scanning range was 4,000–400 cm^−1^ with a resolution of 4 cm^−1^, and the samples were repeatedly scanned 16 times at room temperature.

### 2.9 Scanning electron microscopy

The samples were cut into 3 × 3 × 2 mm flakes and placed in 4% formaldehyde and 2.5% glutaraldehyde mixed solution (1:1, v/v) for two hours. Then, they were washed with a 0.1 M phosphoric acid salt buffer (pH 7.2) 5–10 times, each before being dehydrated with 30%, 40%, 50%, 60%, 70%, 80%, 90%, and 100% (w/w) ethanol solutions for 10 min. Finally, the samples were dehydrated twice with 100% (w/w) acetone for 10 min each time and then freeze‐dried for 15 hr. The dried samples were mounted on a bronze stub and sputter‐coated with gold (Eiko IB‐5; Hitachi). The microstructures were observed using SEM (Nova Nano SEM 230) at a magnification of 500 times.

### 2.10 Fractal analysis of the SEM images

Scanning electron microscopy images were processed with ImageJ 1.50i. The gray level used for thresholding was the median of the gray level histogram of each image before analysis according to Dàvila and Parés (2007). The fractal dimension (D_ƒ_) was calculated using the fractal box counting method, which is given by Equations (2) and (3):

(2) $$D=-\log N_{r}/\log_{r}$$

(3) $$D_{f}=D+1$$

where *N_r_* is the number of boxes at a certain scale containing part of the image; *r* is the corresponding scale; *D* represents the fractal dimension; and *D*_ƒ_ is an extra dimension to actually represent the three‐dimensional features of the gel.

### 2.11 Statistical analysis

The values obtained are expressed as the means, and the error bars indicated the standard deviation. A statistical analysis of variance (ANOVA) of the data was performed using DPS software. A least significant difference (LSD) test with a confidence interval of 95% was used to compare the means. All of the results are expressed as mean ± standard deviation.

## 3 RESULTS AND DISCUSSION

### 3.1 Effect of curdlan content on the water‐holding capacity of myosin gels

**Figure 1:**
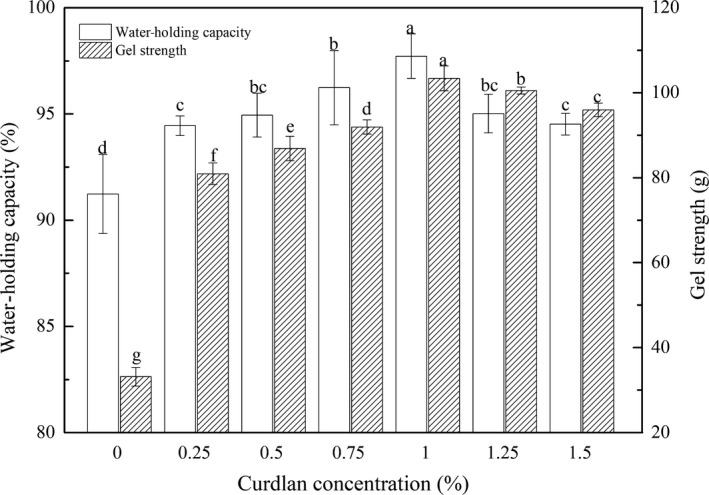
Effect of the different levels of curdlan on the WHC and gel strength of the myosin gel. Different letters denote the significant difference (*p* < 0.05)

As shown in Figure 1, the WHC of the myosin–curdlan mixed gel was higher than that of the control group (a pure myosin gel without curdlan), and the WHC increased with an increasing curdlan concentration within 1%. Hu et al. (2015) found that a noticeable increase in the WHC of fish muscle–curdlan gels (*p* > 0.05) occurred when the curdlan concentration was increased from 0% to 4%. This trend was consistent with the results of this study. Funami et al. (1998) found that curdlan appeared to form a thermally stable, thermo‐irreversible gel within its structure during the heating process at 75°C that entrapped water in the minced pork gel. The addition of curdlan formed denser gels, which could combine with a significant amount of water when heated. Therefore, the curdlan‐added gels could retain more water in the myosin gel network (Chanarat & Benjakul, 2013).

When the concentration of curdlan rose above 1%, WHC decreased. It can be inferred that the higher concentration of curdlan in the myosin retarded the gel‐formation process. Thus, the WHC decreased when the curdlan level rose above 1% (Hu et al., 2015; Wu et al., 2015).

### 3.2 Effect of curdlan content on the gel strength of myosin gels

Figure 1 shows the effect of curdlan content on the gel strength of myosin gels. The highest value of gel strength (103.38 g) was obtained from the gels mixed with 1% curdlan when compared with the control group (33.15 g). As the concentration of curdlan rose above 1%, the gel strength began to decrease. Studies have shown that curdlan expands to form a three‐dimensional network, thus forming a restructured fish meat gel during heating. As a result, the protein matrix network absorbs water and significantly enhances the textural properties of fish products (Funami et al., 1998; Ramírez, Uresti, Velazquez, & Vázquez, 2011). However, excessive curdlan particles may dilute the myosin proteins and disturb gel formation, resulting in decreased gel strength (Hu et al., 2015; Wu et al., 2015).

### 3.3 Effect of curdlan content on the rheological properties of myosin gels

**Figure 2:**
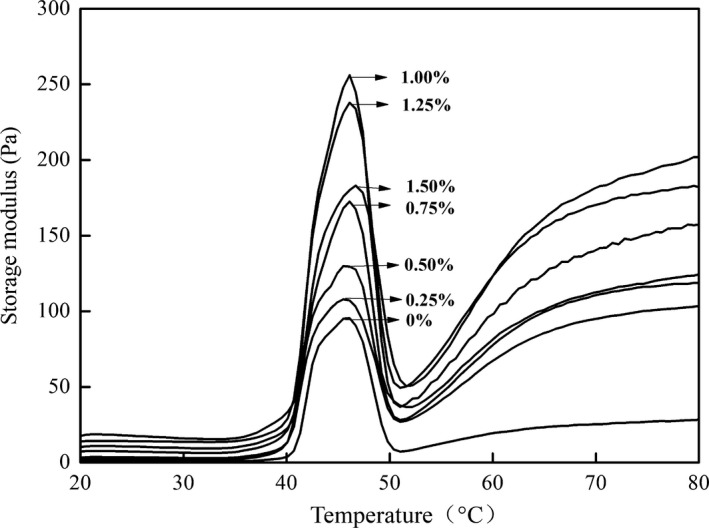
Effect of the different levels of curdlan on the G′ value of myosin during heating

As shown in Figure 2, the G′ of the pure myosin group was almost at the same level when the temperature was below 36.4°C, and began to increase above this temperature. When the temperature increased to 46.1°C, G′ reached the maximum value because the myosin head and hinge portions were denatured and aggregated (Chen, Xu, & Wang, 2007). As the temperature continues to increase, G′ rapidly decreases until 50°C. This phenomenon may be associated with the helix‐to‐coil transition of the myosin, which results in a soaring mobility of the semigel and a disruption of the protein network (Chen et al., 2007; Sano, Noguchi, Tsuchiya, & Matsumoto, 2010). The figure also shows that G′ slowly increases until 80°C. From this result, it may be inferred that the myosin protein underwent further unfolding, aggregation, cross‐linkage, and then formed a more compact three‐dimensional network structure (Sano et al., 2010). Visessanguan, Ogawa, Nakai, and An (2000) and Chen et al. (2007) investigated the changes in the rheological properties of soleus myosin during the heating process and found that there were three different stages in the formation of the gel: (a) the gel‐formation region, (b) the gel‐weakening zone, and (c) the gel‐reinforced area. These findings are consistent with the results of this study.

Figure 2 also illustrates the G′ changes in the myosin–curdlan mixed groups during the heating process and indicates a similar trend for the pure myosin gel. It can be summarized that G′ was the largest at a concentration of 1% curdlan. With the addition of curdlan, the myosin molecules formed a compact structure with a higher G′ value. However, a further increase in the curdlan concentration resulted in a decreased G′ value due to the excessive amount of curdlan rupturing the process of gel formation. The results agree with those of WHC and texture analyses in Sections 3.1 and 3.2.

In summary, the optimum concentration of curdlan in the myosin gel was 1%. The physicochemical properties of the protein gels are related to the moisture distribution and mobility, intermolecular forces, and the microstructure of the gel (Abdelhedi et al., 2016; Zhao et al., 2016). It was important to further investigate how curdlan affected the myosin gel structure. Thus, a 1% concentration of curdlan was chosen to be used in the following examinations.

### 3.4 Proton LF‐NMR relaxation analysis

**Figure 3:**
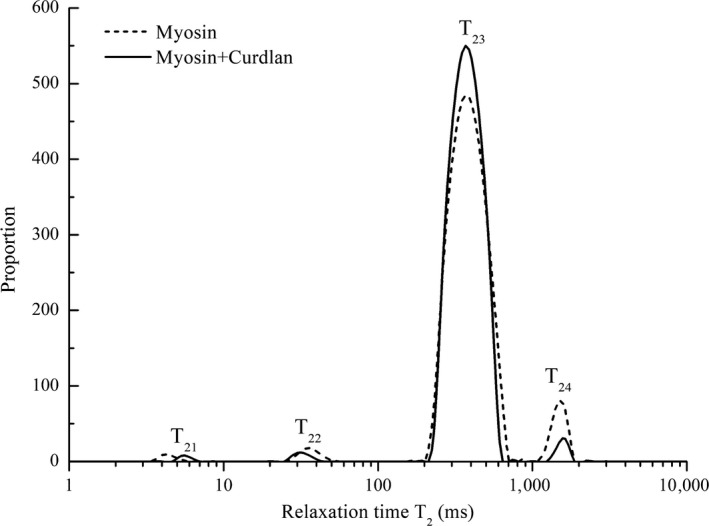
Effect of curdlan on the distribution of the T_2_ relaxation times of the myosin gel

To illustrate the distribution and status of the water in the myosin gel, the LF‐NMR technique characterized by *T*_2_ was employed to investigate water mobility. As shown in Figure 3, the samples with and without curdlan were all characterized using LF‐NMR. Based on the binding pattern and the affinity between water molecules and the material, the water was classified into three kinds (Bertram, Kohler, Böcker, Ofstad, & Andersen, 2006; Chen et al., 2014): *T*_21_ represented the water that binds to the protein in the gel structure (bound water). *T*_22_ and *T*_23_ represent moderately immobile water and immobile water, respectively, and the two can be defined together as immobile water. *T*_24_ represents free water (Goetz & Koehler, 2005; Qin, Xu, Zhou, & Wang, 2015).

Table 1 shows the area percentages (*S*_21_, *S*_22_, *S*_23_, *S*_24_) of the different kinds of water, which also represent their respective populations (Han, Wang, Xu, & Zhou, 2014; Zhang et al., 2016). Table 1 shows that due to the addition of curdlan, the amount of free water (*S*_24_) was reduced from 5.66% to 1.89%, and the content of immobile water (*S*_22_ + *S*_23_) was increased from 93.80% to 97.62%. The sum of the bound water and immobile water (*S*_21_ + *S*_22_ + *S*_23_) increased to more than 98% of the water in the curdlan–myosin mixed gel (Table 1). It can be inferred that curdlan increases the proportion of water tightly bound to a gel network (Han et al., 2014). As a result, the water mobility of the myosin gel decreased with the addition of curdlan.

**Table 1:** *T*_21_, *T*_22_, *T*_23_, and *T*_24_ area percentages of the curdlan–myosin mixed gel

Area percentage (%)	Myosin	Myosin + 1% curdlan
*S*_21_	0.54	0.49
*S*_22_	1.88	1.00
*S*_23_	91.92	96.62
*S*_24_	5.66	1.89

### 3.5 FT‐IR spectroscopy analysis

**Figure 4:**
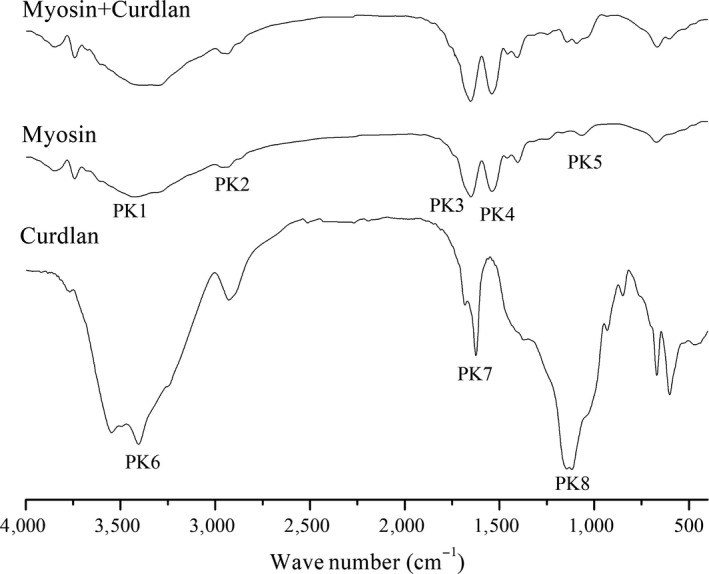
FT‐IR spectra data of the gels

Figure 4 shows the pure myosin and the myosin–curdlan sample, indicated by similar infrared characteristic peaks, including PK1, 3,430 cm^−1^ (amide band A, N–H or O–H stretching vibration peak); PK2, 2,930 cm^−1^; PK3, 1,652 cm^−1^ (amide band I, C=O and C=N stretching vibration peak); PK4, 1,540 cm^−1^ (amide band II, C–N stretching vibration or N–H bending vibration), and PK5, 1,066 cm^−1^ (C–O or C–C stretching vibration; Carbonaro & Nucara, 2010; Perisic, Afseth, Ofstad, & Kohler, 2011). The pure curdlan and the myosin–curdlan sample showed similar absorption peaks for hydrogen bonding hydroxyl groups occurred at PK6, 3,356 cm^−1^ (amide band A, O–H stretching vibration peak) and PK7, 1,636 cm^−1^ (amide band I, C=O stretching vibration peak). The stretching vibration of C–O was PK8 (Jin, Zhang, Yin, & Nishinari, 2006; Wu et al., 2012). This figure indicates there are no novel peaks in the FT‐IR spectroscopy of the mixed gel, and that the addition of curdlan had no effect on the protein structure of the myosin gel or the newly formed group.

Amide A bands are commonly used to analyze the interaction between protein molecules and water molecules (Andreeva & Karamancheva, 2001; Ma et al., 2013). The addition of curdlan allowed PK1 and PK6 shift to a lower wave number (3,312 cm^−1^), indicating that the role of hydrogen bonds was enhanced by the addition of curdlan in the formation of protein gels (Barth, 2007; Chen et al., 2014), thus increasing the gel strength and WHC. In minced pork gel, Funami et al. (1998) found that the meat–curdlan mixed gel heated at 75°C with increased ionic strength formed a high‐set gel with improved heat‐stable properties, which showed similar properties to those of the 90°C mixed gel. This could be because the formation of hydrogen bonds was inhibited by salts rather than myosin when cross‐linked in the junction zones. This is inconsistent with our results. This discrepancy may be due to the difference in ionic strength and materials.

### 3.6 Microstructure

**Figure 5:**
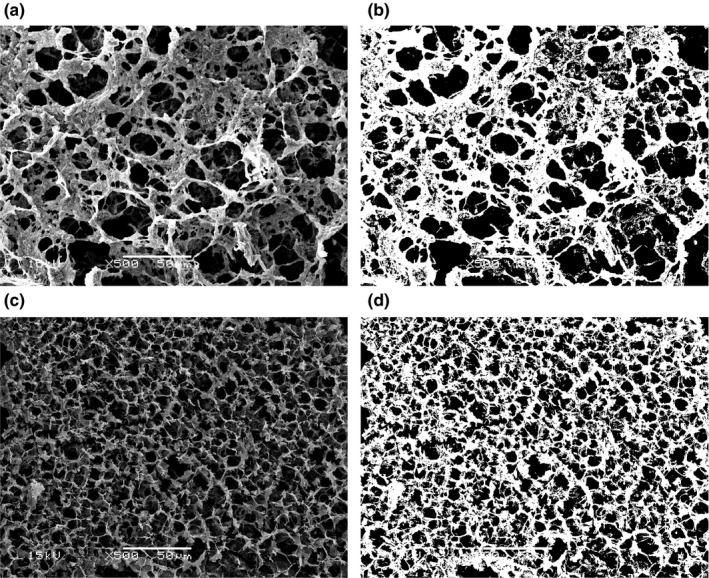
SEM of the gels (left) and the corresponding binary images (right) formed from the myosin gel: (a) pure myosin and (b) myosin + curdlan

To confirm the assumption that the myosin formed a more compact gel structure with the addition of curdlan, the microstructure of gel samples was observed by SEM. It was found that the pure myosin group showed a gel network with large pores, as well as a coarse and scattered structure (Figure 5a). Moreover, a denser gel structure with small and evenly distributed pores was formed by adding the curdlan (Figure 5b). The D_ƒ_ of the myosin–curdlan mixed gel was increased when compared with the pure myosin gel (from 2.82 to 2.84), which indicated a tight cross‐linking in the gel network structure (Bi, Li, Wang, & Adhikari, 2013). As a result, an impact and uniform gel structure was formed (Balange & Benjakul, 2009). This result verified that curdlan enhanced the physicochemical properties of the myosin–curdlan mixed gel when compared with that of the pure myosin gel.

## 4 CONCLUSIONS

The gel strength and WHC of the myosin gel increased with the addition of curdlan. Moreover, intermolecular hydrogen bonding was enhanced and a denser structure with small pores was formed, hindering the liquidity of water in the mixed gels and generating enhanced physicochemical properties. Thus, it may be concluded that curdlan can be used as an additive to significantly improve the physicochemical properties of myosin gels and the quality of surimi‐based seafood products.

## CONFLICT OF INTEREST

All authors declare that there is no conflict of interest.

## ETHICAL STATEMENT

There was no human or animal testing in this study.
